# Supplementary material for: Collaborative intelligence in AI: Evaluating the performance of a council of AIs on the USMLE
Source: PLOS Digit Health. 2025 Oct 9;4(10):e0000787. doi: 10.1371/journal.pdig.0000787 (PMC12510544; doi:10.1371/journal.pdig.0000787)
Supplement: S2 File — (ZIP) [file pdig.0000787.s002.zip › S2 Data Raw data files containing unprocessed question inputs and LLM outputs including transcripts of deliberations/STEP3_Question-66-1_Type-MC-NJ.docx]

**Facilitator** **[Round 1]:** Essepro™ (lesystolol) Reduces Blood Pressure Significantly No significant interactions with commonly used medications were observed. No significant interactions with hydrochlorthiazide, furosemide, losartan orlisinopril. No significant interactions with digoxin, warfarin or simvastatin. Drugs that inhibit CYP2C9 can increase plasma levels of Essepro. Patients on Essepro who are also treated with drugs that inhibit or induce this enzyme shhould be monitored closely, and dosage of Essepro may need to be adjusted based on blood pressure response. Important Safety Information Patients treated with Essepro should be advised against sudden discontinuation of therapy. When discontinuing therapy, dosage should be gradually tapered over 2 weeks. Essepro is contraindicated i patients with bradycardia, heart block greater than first degree, cardiogenic shock, decompensated cardiac failure, severe hepatic impairment, and in patients who are hypersensitive to any component of this product. Essepro should be used with caution in patients with peripheral vascular disease, renal impairment or thyrotoxicoses. Caution should be used in diabetics, as beta blockers may mask some manifestations of hypoglycemia. In general, patients with bronchospastic disease should not receive beta blockers. A 65-year-old woman comes to the office for blood pressure medication management. Medical history is significant for poorly controlled hypertension, psoriasis, and psoriatic arthritis previously treated with methotrexate. Additional medical history is significant for alcohol use disorder and elevated liver function tests. Medications include enalapril, spironolactone, and topical corticosteroids. Vital signs are normal except for a blood pressure of 160/104 mm Hg. Physical examination discloses thick, scaly plaques on the scalp, buttocks, and upper and lower extremities. There are several spider angiomata on the chest and abdomen. The abdomen is distended and a fluid wave is noted. She has 2+ lower extremity edema. The patient says she would like to try a new drug called Essepro to treat her hypertension because she can get a 3-month supply of the medication for free. 66. Which of the following is the most appropriate response to the patient's request for the medication? (A) Essepro should be prescribed because she can get it for free (B) Essepro should not be prescribed because it can worsen her psoriasis (C) Essepro should not be prescribed because it is similar to her other medications (D) Essepro should not be prescribed because the patient has severe liver disease (E) Essepro should only be used for hypertensive emergencies

**AI_D:** RESTATING THE QUESTION: This query pertains to the appropriateness of prescribing Essepro, a beta blocker, to a 65-year-old woman who has a series of medical conditions such as poorly managed hypertension, psoriasis, psoriatic arthritis, alcohol use disorder, and abnormal liver function. Beta blockers are a class of medications used to manage hypertension by decreasing the heart rate and reducing the contractility of the heart. They also inhibit the activity of specific enzymes, leading to interactions which may require monitoring and dosage adjustment. PLANNING: Several factors need to be examined when contemplating the use of Essepro. These include its potential interactions with existing medications; the character of the patient's current medical conditions, in particular, her hypertension and liver function; the potential side effects of Essepro; and the financial benefits the patient is expecting. CLINICAL REASONING: The USFDA contraindicates the use of Essepro in patients with severe hepatic impairment, like our patient. Her severe liver disease, elevated liver function tests, alcohol use disorder, presence of spider angiomata, distended abdomen with fluid wave, and 2+ lower extremity edema all add up to a situation of decompensated liver cirrhosis. This condition can further get complicated into conditions such as ascites, portal hypertension etc., which would complicate the patient's health status. Your next point under observation relates to her poorly managed hypertension. Since Essepro is a beta blocker, it might help manage the high blood pressure. However, her overall health status and in particular the severity of her liver disease makes the prescription of this drug less advisable. CONCLUDING: Given the patient's significant liver disease, contra-indicative for the use of Essepro, and, given the severity of her poorly controlled hypertension and alcohol use disorder, prescribing Essepro would likely be an inappropriate action despite the possible financial benefits for the patient. RESPONSE: So, option (D) "Essepro should not be prescribed because the patient has severe liver disease" seems the most appropriate response regarding the patient's request for the medication.

**AI_B:** RESTATING THE QUESTION: The patient, a 65-year-old woman, presents for the management of her blood pressure medication. She suffers from hypertension, psoriasis, psoriatic arthritis, alcohol use disorder, and her liver function tests are elevated. Her current medications include enalapril, spironolactone, and topical corticosteroids. She is considering trying a new drug called Essepro for her hypertension, mainly because she can obtain a three-month supply of the medication for free. The query is essentially about what the best response to her request for Essepro should be, taking into account her current health status and co-morbidities. PLANNING: Given the patient's liver condition, any new medication prescribed should not exacerbate it. Essepro, as stated, is contraindicated in patients with severe hepatic impairment, which could be related to the patient's alcohol use disorder and elevated liver function tests. Therefore, there is an assumption here that Essepro might be unsuitable because of her liver condition. CLINICAL REASONING: The statement provided describes that the patient's medical history includes substantial alcohol misuse and she has elevated liver function tests, indicating liver dysfunction. Essepro is contraindicated in severe hepatic impairment. With these considerations, it is reasonable to consider that the patient's liver disease can be severe, making Essepro an inappropriate choice. CONCLUDING: From a clinical viewpoint, the patient's liver damage could potentially be worsened by the introduction of Essepro. While the economic argument for prescribing it due to its zero-cost availability is valid, it is critical to put the patient's health first. The primary concern should be the safety of the patient, keeping in mind her alcohol use disorder and elevated liver function tests. RESPONSE: Hence, (D) Essepro should not be prescribed because the patient has severe liver disease is the most appropriate response to the patient's request for the medication.

**AI_A:** (1) RESTATING THE QUESTION: Here's the issue under discussion: A 65-year-old woman is struggling to control her hypertension and wants to try a new hypertension medication called Essepro. She's drawn to it because she can obtain a three-month supply of Essepro free of charge. Concomitantly, the patient has severe psoriasis and psoriatic arthritis. She also has a record of alcohol use disorder and elevated liver function tests. Physically, she exhibits signs of high blood pressure and various symptoms associated with liver disease. We have to decide the most viable response to her request to start taking Essepro, given her current health condition. (2) PLANNING: This decision requires analysis of a few assumptions: Assumption 1) Essepro is a safe and effective hypertension medication for her, particularly in relation to her other current treatments, assumption 2) the cost and availability of Essepro are the most important factors to consider, assumption 3) the drug's impact on her psoriasis and liver health will not be harmful, and assumption 4) her severe liver disease is not a contraindication to taking Essepro. Among these assumptions, the fourth - the effect of Essepro on her liver disease - is clinically the most critical to scrutinize. (3) CLINICAL REASONING: Essepro is established to be a medication that significantly reduces blood pressure, which appears to suit her needs. From the provided information, it does not have any significant interactions with commonly used hypertension medications including enalapril, the drug she is currently on. In contrast, it is important to note that Essepro is contraindicated in patients with severe hepatic impairment. This means it is not recommended for patients with serious liver disease because it might worsen their condition or cause other dangerous side effects. From her history, it is evident that our patient has a potential liver disorder evidenced by her elevated liver function tests and physical manifestations including the distended abdomen and spider angiomata, signs of portal hypertension. Our discussion, therefore, should pivot from the fourth assumption that her severe liver disease is not a contraindication to taking Essepro. (4) CONCLUDING: Given her reported alcohol use disorder and apparent signs of liver disease, we now have to consider this in our patient's treatment planning. If we were to prescribe Essepro disregarding this factor, the consequences could be severe. The patient's liver health may further deteriorate, contributing to an escalation in her disease. As practitioners, we should prioritize her overall well-being over the temporary advantage she may obtain from receiving a three-month free supply of Essepro. (5) RESPONSE: Consequently, the most fitting response to the patient's request for the medication is (D) Essepro should not be prescribed because the patient has severe liver disease. This decision is based on prioritizing her safety and overall health. The primary goal is to manage her hypertension effectively without exacerbating her existing liver disease.

**AI_E:** RESTATING THE QUESTION: The question presents a 65-year-old woman with a medical history of poorly controlled hypertension, psoriasis, psoriatic arthritis, alcohol use disorder and elevated liver function tests. She is currently on medications including enalapril, spironolactone, and topical corticosteroids. On examination, she has significant features suggestive of chronic liver disease with physical signs of portal hypertension. Due to elevated blood pressure, she wants to start a new medication, Essepro, as she can obtain it for free. The task is to determine the most suitable response to this patient's request. PLANNING: Looking at the given options, we can tentatively sort them into categories based on their inherent assumptions: patient's economic condition (A), potential drug-disease interaction (B), similarity to existing drugs (C), patient's current comorbidity (D), and usage restrictions of the drug (E). Let's analyse these assumptions. 1. A key consideration in managing a patient's health is their ability to afford prescribed medications. While this is important, prescribing a medication should primarily be based on its therapeutic benefits and compatibility with the patient's current health status; the cost factor should be a secondary consideration. 2. The potential of Essepro to worsen the psoriasis should be evaluated. 3. Determining whether Essepro is similar to her existing medications requires an analysis of their mechanisms of action, side effect profiles, and drug interactions. If they are similar, it would be redundant to add Essepro to her regimen. 4. Her significant history of liver disease should be taken into account, especially given the known contraindication in Essepro's product information: "severe hepatic impairment." 5. Lastly, the usage of Essepro for hypertensive emergencies distinctly insinuates that this drug may not be suitable in the management of long-term hypertension. CLINICAL REASONING: Essepro is a beta-blocker used to manage hypertension with no significant interactions with the patient's current medications. However, it is contraindicated in severe hepatic impairment. The patient's physical examination showing spider angiomata, abdominal distension with a fluid wave (indicative of ascites), and lower extremity edema suggest chronic liver disease and portal hypertension, signs of substantial liver function impairment. With such clues, it is likely the elevated liver function tests indicated in her medical history represent more than just a mild disease. Therefore, it could be assumed that the patient has severe hepatic impairment. CONCLUDING: The use of Essepro is contraindicated in severe hepatic impairment. Given the patient's clinical history and examination findings, there is strong evidence to suggest advanced liver disease. Thus, prescribing Essepro to this patient could potentially be harmful. RESPONSE: The most appropriate response to the patient's request for Essepro would be option (D) Essepro should not be prescribed because the patient has severe liver disease.

**AI_C:** (1) RESTATING THE QUESTION: This query wants to determine the most suitable response to a 65-year-old patient's request to try Essepro, a blood pressure medication, as a potential remedy for her poorly controlled hypertension. In addition to hypertension, the individual is affected by psoriasis, psoriatic arthritis, and has an alcohol misuse disorder with elevated liver function test results. Her medication regimen currently consists of enalapril, spironolactone, and topical corticosteroids. She presents with some physical signs associated with both her skin condition and advanced liver disease. Essepro is a drug known for effectively lowering blood pressure without causing significant interactions with many commonly prescribed medications. However, this drug can pose problems in people with bradycardia, heart block beyond first degree, cardiogenic shock, decompensated cardiac failure, severe liver impairment, and hypersensitivity to any component of the drug. (2) PLANNING: The primary assumptions made here are the patient's poor blood pressure control, the observation of physical signs indicating elevated liver function, and her request for Essepro, mandating a careful consideration of possible drug interactions and contraindications. Alternate assumptions that could be made are that her current medications are not able to adequately control her hypertension, suggesting a need for a different approach; or her liver function test results and physical signs may indicate a severe form of liver disease, which could be a contraindication for Essepro. Clinically, the most appropriate assumption to pursue would be the latter, given the contraindication specifically listed for Essepro in individuals with severe liver impairment. (3) CLINICAL REASONING: To determine the best course of action, an assessment of the patient's overall health, potential for drug interactions, and current disease state is needed. The patient's high blood pressure is currently poorly managed, however the introduction of Essepro may not be the best step. While Essepro is successful in reducing blood pressure, it is also cautioned against in cases of severe liver impairment as per the provided drug information. The patient's elevated liver function tests as well as physical signs such as spider angiomata, distended abdomen, and a fluid wave all indicate a higher likelihood of liver impairment. Given the additional medical history of alcohol use disorder, it could be inferred that this patient may potentially have a form of severe liver disease. Introducing Essepro in such a case could exacerbate existing complications, therefore it's essential we verify the severity of this patient's liver condition before making a decision. (4) CONCLUDING: Clinical judgment is exercised here to infer that treating the patient's hypertension is important but should not compromise her overall health. In this particular scenario, the use of Essepro may have significant adverse effects due to the patient's severe liver impairment based on the physical features and her medical history, outweighing its potential benefits. (5) RESPONSE: Therefore, the most appropriate response to the patient's request for medication would be (D) Essepro should not be prescribed because the patient has severe liver disease.

**Facilitator** The consensus statement synthesizing the responses of AI_A, AI_B, AI_C, AI_D, and AI_E is as follows: The question at hand pertains to a 65-year-old woman with a complex medical history, including poorly controlled hypertension, psoriasis, psoriatic arthritis, alcohol use disorder, and elevated liver function tests. She is currently on medications including enalapril, spironolactone, and topical corticosteroids. She has expressed interest in trying a new hypertension medication, Essepro, primarily due to its cost-effectiveness as she can obtain a three-month supply for free. The primary considerations in this scenario are the patient's overall health, the potential interactions of Essepro with her current medications, and the contraindications of Essepro. While Essepro is known to effectively reduce blood pressure and does not have significant interactions with commonly used hypertension medications, it is contraindicated in patients with severe hepatic impairment. The patient's medical history and physical examination suggest the presence of severe liver disease, which is a contraindication for Essepro. Despite the financial benefits of Essepro, the patient's health should be the primary concern. Given her significant liver disease, prescribing Essepro could potentially exacerbate her condition and lead to severe consequences. Therefore, the most appropriate response to the patient's request for Essepro is option (D) "Essepro should not be prescribed because the patient has severe liver disease". This decision prioritizes the patient's safety and overall health, aiming to manage her hypertension effectively without worsening her existing liver disease.
